# Probiotics, Prebiotics, Synbiotics, and Postbiotics Against Oral Candida in Children: A Review of Clinical Trials

**DOI:** 10.3390/nu17142253

**Published:** 2025-07-08

**Authors:** Anna Turska-Szybka, Dorota Olczak-Kowalczyk, Svante Twetman

**Affiliations:** 1 Department of Paediatric Dentistry, Medical University of Warsaw, Żwirki i Wigury St. 61, 02-091 Warsaw, Poland; 2 Department of Odontology, Faculty of Health and Medical Sciences, University of Copenhagen, Denmark Nørre Allé 20, 2200 Copenhagen, Denmark; stwe@sund.ku.dk

**Keywords:** dental caries, children, fungus, *Candida* spp., *Lactobacillus*, *Streptococcus*

## Abstract

Background/Objectives: Children with an oral presence of *Candida* spp. have an elevated prevalence of dental caries. As an alternative to conventional antifungal drugs, the use of biofilm-modulating strategies, such as probiotic bacteria, may be a sustainable option. Probiotics are live microorganisms that have beneficial health effects, while prebiotics are compounds in food that foster the growth or activity of the beneficial microorganisms. The aim of this paper was to review current clinical findings regarding the antifungal effects of pre- and probiotic supplements, including syn- and postbiotics, in children. Methods: We searched two databases (PubMed and Google Scholar) for controlled clinical trials published in English up to 20 April 2025, and two authors scanned the abstracts independently for relevance. The selected full-text papers were reviewed and assessed for risk of bias. Results: Four articles published between 2013 and 2025 were included in this review, covering a total number of 208 caries-active children between 3 and 14 years of age. Study designs were heterogeneous, and we observed conflicting results: two studies with probiotic streptococci failed to demonstrate any beneficial effects on the counts of salivary *C. albicans*, while interventions with *L. plantarum* and *L. rhamnosus* significantly reduced *C. albicans* compared with controls. None of the included reports displayed a low risk of bias. No clinical studies utilizing prebiotics, synbiotics, or postbiotics were retrieved. Conclusions: We found insufficient evidence concerning the antifungal effects of probiotic supplements in children. Therefore, we recommend future clinical trials to explore the ability of pre-, pro-, and postbiotic interventions to affect cross-kingdom biofilms in order to support a balanced and health-associated composition of the dental biofilm in children.

## 1. Introduction

Dental caries is a common non-communicable disease, affecting a significant part of the global population [1]. According to current estimates, early childhood caries affects 48% of all preschool children, ranging from 30% in Africa to 82% in Oceania [2]. The disease has a complex etiology with a mix of genetic, dietary, social, lifestyle, and behavior-related causal components, leading to a dysbiotic overgrowth and an abundance of acidogenic and aciduric bacteria in the dental biofilm [3,4]. In particular, microbial cross-kingdom communities in the oral environment—with a partnership between bacteria and fungi—are associated with an increased risk of caries development [5]. Consequently, children with the presence of *Candida* spp. have a higher prevalence of dental caries when compared to children without these microorganisms [6,7,8]. *Candida albicans* is a highly acidogenic opportunistic oral pathogen commonly identified in preschool children [8]. The yeast does not colonize teeth but adheres to the oral mucosa and interacts with *Streptococcus* spp., which results in an increased accumulation of *Streptococcus mutans* and an elevated production of extracellular matrix in the dental biofilm [7,9]. These properties suggest that the prevention of Candida colonization may contribute to arrest caries development in young children. The common conventional oral antifungal drugs (nystatin, miconazole, and fluconazole) are certainly effective but may be associated with side effects, such as discomfort, sores, taste changes, nausea, vomiting, diarrhea, and abdominal pain. Drug interactions may also occur. Therefore, safer and more sustainable biofilm-modulating strategies have gained interest over the past two decades, in particular the use of prebiotics and probiotics [10,11,12]. Probiotics are live bacteria that confer a health benefit on the host when taken in sufficient amounts [13], whereas prebiotics are food substrates that enhance the growth and activity of probiotic bacteria [14]. Postbiotics (sometimes called paraprobiotics) are inactivated probiotic bacteria, their metabolites, or extracted cell walls, while synbiotics are a combination of prebiotics and probiotics [15]. The effect of prebiotic and probiotic supplements on oral candida in vivo has been covered in systematic reviews in recent years [16,17,18]. The studies reporting positive outcomes were, however, conducted among elderly individuals categorized as frail [19,20,21] or denture wearers [22], while information on antifungal effects in young children were lacking [23]. This merits a literature update, and the aim of the present paper was to review current clinical findings regarding the antifungal effects of pre- and probiotic supplements, including syn- and postbiotics, in children. The specific research question was as follows: Do “biotic” supplements reduce the oral counts of *Candida albicans* in children?

## 2. Materials and Methods

We searched two databases (PubMed and Google Scholar) up to 20 April 2025 in order to retrieve the relevant literature. The main search terms in PubMed were (“probiotics” OR “probiotic” OR “prebiotics” OR “prebiotic” OR “synbiotics” OR “synbiotic” OR “postbiotics” OR “postbiotic”) AND (“oral candidiasis” OR “oral candidiases” OR “oral candida” OR “thrush”) AND (“infants” OR “children” OR “adolescents”). Randomized or non-randomized clinical trials published in English were considered using the following PICO: Population: Preschool children, schoolchildren, and adolescents <18 years of age; Intervention: Oral exposure/intake of probiotics, prebiotics, synbiotics, or postbiotics of any type and dosage; Control: Any placebo/control treatment or “treatment as usual”; Outcome: *Candida* spp. counts/carriage in samples of saliva or oral/dental biofilm.

For initial screening of relevance, we read the abstracts of the listed papers. When in doubt, we ordered full-text versions and two authors reviewed them independently. We checked systematic and narrative reviews for background information and additional references of potential interest. Studies with fewer than 10 participants, case reports, academic theses, and the grey literature were not included. Furthermore, we excluded publications describing studies in animals, in vitro studies, or ex vivo experiments based on clinical samples. Two authors (A.T.-S.; S.T.) extracted key data from the eligible and selected papers and assessed the risk of bias (low, moderate, high) according to the *Cochrane Handbook for Systematic Reviews of Interventions* [24]. We solved disagreements with a consensus discussion. The main findings concerning study design, sample size, intervention and duration, control, and results are presented descriptively.

## 3. Results

### 3.1. Included Papers

From a list of over 300 papers, four articles published between 2013 and 2025 were included in this review [25,26,27,28]. The articles covered a total number of 208 children, 3–14 years of age. Countries of origin were New Zealand [25], India [26,27], and China [28]. The main characteristics, along with the authors’ conclusions, are shown in Table 1.

The interventions consisted of probiotic bacteria belonging to the *Lactobacillus* (n = 2) or *Streptococcus* (n = 2) genera with twice-daily exposures (mouthwashes or powder) and durations ranging from one week to three months. The doses of active probiotic bacteria varied from 10^8^ to 2 × 10^9^ colony-forming units (CFU) per day. The studied populations consisted of children with active caries or children who had recently received a comprehensive preventive and restorative caries treatment [27]. Three studies relied on the cultivation of salivary candida on selective agar plates, while the endpoint was based on 18S sRNA gene sequencing in one study [28]. No studies utilizing interventions with prebiotics, synbiotics, or postbiotics were identified.

### 3.2. Main Findings

The study designs were heterogeneous, and the results were conflicting. Two studies reported on the effect of lozenges/tablets containing probiotic streptococci in caries-active schoolchildren vs. placebo [25] or herbal mouthrinse (Tulsi licorice) [26]. Both studies failed to demonstrate any significant effects on the counts of salivary *C. albicans* in comparison with the control groups. The two most recent studies tested probiotic lactobacilli against chlorhexidine mouthwash in children with Down syndrome [27], and against placebo in preschool children with early childhood caries [28]; the interventions lasted 2 and 4 weeks, respectively. The results from both studies were clear-cut and encouraging: *L*. *plantarum* CCFM8748 significantly reduced the amounts of *C. albicans* in saliva compared with placebo [28]; and the test group rinsing with suspensions containing *L. rhamnosus* GG demonstrated significantly lower *C. albicans* counts than the chlorhexidine-rinsing group [27]. No harmful side effects or adverse effects of a serious nature were described in any of the papers.

### 3.3. Risk-of-Bias Assessment

The assessed risk of bias is shown in Table 2. None of the retrieved and included studies had a low risk of bias.

## 4. Discussion

The role of *Candida albicans* in creating and maintaining a cariogenic biofilm is well established [11,29]. Candida is a powerful opportunistic yeast that relies on the production of short-chain carboxylic acids and proteases, with a strong ability to form biofilms and promote the growth of *S. mutans* [10]. Previous research in vitro has shown that probiotic bacteria have an anti-biofilm effect against *C. albicans* [30,31,32], and ex vivo in clinical isolates sampled from children with active caries [33,34]. The proposed mechanisms of action are multiple: probiotic *Lactobacillus* species produce acids or exometabolites that inhibit *C. albicans* growth [35]; they downregulate critical genes for biofilm formation [29]; and they reduce the expression of hyphal-related genes [36]. In addition, prebiotic arginine may counteract biofilm acidification and thereby suppress the outgrowth of opportunistic pathogens, such as *Candida* spp. [37]. A study in vitro has also demonstrated that prebiotic xylitol, a common ingredient in oral care products, can inhibit growth of C. albicans together with lactobacilli isolated from the oral cavity [38].

Most previous reports on the present research topic have utilized probiotic lactobacilli for evaluating the antifungal effects in vitro and in vivo. Therefore, it was not surprising to find beneficial results in the two clinical trials that investigated the impact of *L. rhamnosus* and *L. plantarum*, respectively. This observation was also in accordance with clinical studies in adults, in which reductions in oral *Candida* carriage rates were obtained by probiotic *Lactobacillus* strains, alone or in combination with *Bifidobacterium* [19,20,21,22]. There is evidence suggesting that the efficacy of probiotics is genus-, strain-, and disease-specific [39], and this can likely explain the different results of the selected studies rather than methodological issues. Certainly, this does not necessarily mean that probiotic streptococci lack an effect on *Candida* growth in the dental biofilm but that remains to be further investigated. As an example of this complexity, the ProBiora3 product used by Mishra et al. [26] did not seem to affect the oral candida colonization but proved to be effective in the prevention of early childhood caries [40].

Due to the limited number of eligible papers, no further analyses and conclusions concerning optimal doses, the mode of administration, and the duration of interventions were possible, and these issues remain knowledge gaps. The highest daily dosage unveiled in this review was two billion CFU, which possibly was suboptimal in comparison with internal medicine; according to a prior systematic review, a dosage of at least 10^10^ CFU per day was required to colonize the digestive tract and to treat acute gastroenteritis in children [41]. An important but somewhat expected finding was that none of the trials reported adverse events of a serious nature in connection with the interventions. The products investigated contained bacterial species or strains that were labeled “Generally Recognized as Safe” (GRAS).

Previous clinical studies have presented emerging evidence of low certainty that lozenges/tablets containing synbiotics or postbiotics could reduce caries incidence in preschool and schoolchildren in comparison with placebo and standard preventive care [42]. However, we found no clinical papers on the antifungal effects of prebiotics, synbiotics, and postbiotics in the oral cavity. This was notable since there are in vitro studies showing antifungal effects of prebiotic arginine [37] and xylitol [43]. Obviously, prebiotics may hold the power to manipulate the diseased oral microbiome [44], and the rationale to use them in oral care products is that they present a safe alternative to probiotic interventions and are less susceptible to environmental factors. Synbiotics and postbiotics largely share similar antifungal modes of action with probiotics, including the production of acids, hydrogen peroxide, and bacteriocins [45]. They may also compete for adhesion sites with caries-associated bacteria and modulate the immune response [44]. In this context, postbiotics may be of particular interest for both producers and consumers since they have a better thermal stability, ease of storage, and longer shelf-life [46]. This makes them suitable for addition to already existing oral care products, such as toothpaste and rinses, which could facilitate their use. Furthermore, postbiotic supplements traditionally rely on heat-inactivated bacteria or cell-free suspensions but there are more technologies available, such as ultrasonication, magnetic technologies, and plasma technologies [10].

We searched only the literature published in English and in only two different databases, which were possible shortcomings of this review. Due to the limited number of studies, their methodological shortcomings, and inconsistent results, we consider the current evidence for a possible antifungal capacity of probiotics, prebiotics, synbiotics, and postbiotics in childhood insufficient. Unreliable—or lack of—evidence is, however, not the same as a lack of effect. The first 1000 days of life are a critical window for the influence of environmental exposures on the assembly of the oral microbiome, which is the precursor to dental caries [47]. In this context, this period in childhood provides a unique opportunity for shaping the oral microbiota through ecological interventions to combat dysbiosis. Thus, the role of all “biotics” in affecting the presence of oral candida in childhood warrants further clinical investigation. In this context, clinical trials with enough power, employing standardized interventions and analytical methods, and reporting a core outcome set would be desirable.

## 5. Conclusions

In this review, we found insufficient evidence concerning the effect of probiotic bacteria on oral candida colonization in children. No clinical studies utilizing prebiotic, symbiotic, or postbiotic supplements were identified. Due to this knowledge gap, we recommend future clinical trials to explore the effectiveness of various “biotics” in affecting cross-kingdom biofilms and their capacity to maintain dental biofilm symbiosis in children.

## Figures and Tables

**Table 1:** Main characteristics and outcome of the included studies.

First Author, Year, Country, [Ref.]	Study Design	Study Group	Intervention; Number (n)	Control; Number (n)	Duration of Intervention	Endpoint	Main Result
Burton, 2013, New Zealand, [25]	RCT	Schoolchildren with active caries, 5–10 yrs	Two lozenges per day with *S. salivarius* M18 ^a^; n = 40	Placebo lozenges; n = 43	3 months	Candida counts in saliva, cultivated onto Chromogenic agar	No significant difference between the groups but detailed
Mishra, 2016, India, [26]	RCT	Schoolchildren with caries, 6–14 yrs	Probiotic tablet (ProBiora3 ^b^) in water (mouthwash); n = 20	Herbal gargle; n = 20	1 week	Candida counts in saliva, cultured onto Sabouraud agar plates	No significant difference between the groups
Saha, 2025, India, [27]	RCT	Schoolchildren with Down syndrome after full oral rehabilitation, 6–14 yrs	Probiotic mouthwash with *L. rhamnosus* GG ^c^; n = 15	Chlorhexidine gluconate mouthwash (0.2% *w*/*v*); n = 15	2 weeks	Candida counts in saliva, cultured onto Sabouraud agar plates	Significantly lower counts in the test group: median Log_10_ values 1.0 vs. 2.0 after 2 weeks; 0.0 vs. 1.0 at 6-month follow-up
Zhang, 2023, China, [28]	RCT	Children with caries, 3–6 yrs	Probiotic powder with *L. plantarum* ^d^ in maltodextrin (2 g); n = 29	Placebo powder in maltodextrin (2 g); n = 26	4 weeks	Candida amount in saliva (18S sRNA)	Significantly reduced in the test group with a mean decrease of 0.11 log copies/ng DNA; no changes in the placebo group

^a^ 10^9^ CFU per lozenge; ^b^ blend of *Streptococcus uberis* KJ2, *Streptococcus oralis* KJ3, and *Streptococcus rattus* JH145, 10^6^–10^8^ CFU of each strain in the tablet; ^c^ bacterial count: 8 × 10^9^ CFU/g; ^d^ saline suspension with 10^8^ CFU (30 mg powder).

**Table 2:** Risk-of-bias assessment.

First Author, Year [Reference]	Selection Bias	Allocation Concealment	Blinding of Performance Bias	Attrition Bias	Selective Reporting	Other Bias
Burton, 2013 [25]	⊕	⊕	⊕	⊕	⊕	⊕
Mishra, 2016 [26]	⊕	⊕	⊕	⊕	⊕	⊕
Zhang, 2023 [28]	⊕	⊕	⊕	⊕	⊕	⊕
Saha, 2025 [27]	⊕	⊕	⊕	⊕	⊕	⊕

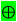
 = green: low risk of bias; 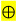 = yellow: moderate or unclear risk of bias; 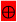 = red: high risk of bias.

## Data Availability

No new datasets were generated during the current study.

## Abbreviations

The following abbreviations were used in this manuscript:

CFU	Colony-forming units
RCT	Randomized controlled trial
